# Periodontitis in elderly patients with type 2 diabetes mellitus: impact on gut microbiota and systemic inflammation

**DOI:** 10.18632/aging.202174

**Published:** 2020-11-25

**Authors:** Jinyou Li, Haifeng Lu, Huanwen Wu, Shunmei Huang, Lufang Chen, Qifeng Gui, Wenjing Zhou, Yichen Yang, Yue Wu, Hua Zhang, Qin Zhang, Yunmei Yang

**Affiliations:** 1 Zhejiang Provincial Key Laboratory for Diagnosis and Treatment of Aging and Physic-Chemical Injury Diseases, The First Affiliated Hospital, School of Medicine, Zhejiang University, Hangzhou, Zhejiang, China; 2 State Key Laboratory for Diagnosis and Treatment of Infectious Disease, National Clinical Research Center for Infectious Diseases, Collaborative Innovation Center for Diagnosis and Treatment of Infectious Diseases, The First Affiliated Hospital, College of Medicine, Zhejiang University, Hangzhou, Zhejiang, China; 3 Deanery of Biomedical Sciences, The University of Edinburgh, Edinburgh, UK

**Keywords:** periodontitis, gut microbiota, older individuals, diabetes, systemic inflammation

## Abstract

Elderly patients with type 2 diabetes mellitus (T2DM) exhibit considerable periodontitis frequency, which causes tooth loss and poor quality of life. To investigate the impact of periodontitis on gut microbiota, we used 16S rRNA amplicon sequencing to characterize the composition and structure of gut microbiota among elderly patients with T2DM and periodontitis (T2DM_P), elderly patients with T2DM alone (T2DM_NP), and healthy volunteers. We identified 34 key gut microbiota markers that distinguished participants with different periodontal conditions and investigated their connections to other gut bacteria, as well as their clinical correlates. The most striking differences in co-occurrence networks between the T2DM_P and T2DM_NP groups comprised interactions involving dominant genera in the oral cavity (i.e., *Streptococcus* and *Veillonella*). Of the 34 identified key gut microbiota markers that distinguished participants with different periodontal conditions, 25 taxa were correlated with duration of diabetes, dry mouth or the peripheral levels of pro-inflammatory cytokines (e.g., tumor necrosis factor-α, interferon-γ, prostaglandin E2, interleukin-17, and interleukin-6) and metabolic parameters (e.g., hemoglobin A1c), respectively. Our findings suggest that gut microbial shifts driven by periodontitis may contribute to systemic inflammation and metabolic dysfunction during the progression of T2DM.

## INTRODUCTION

The number of elderly individuals with type 2 diabetes mellitus (T2DM) has dramatically increased worldwide in the past few decades [1]. Periodontitis is a common oral complication of T2DM among elderly individuals, such that it affects more than half of patients with T2DM [2]. There is considerable evidence regarding the substantial influence of periodontitis in malnutrition, disability, and reduced quality of life in older adults; however, the underlying mechanisms remain poorly understood [3]. Growing interest in the complex interaction between oral and gut bacteria has resulted in the identification of appreciable numbers of oral taxa in the gut microbiota of adults regardless of periodontal status [4]. In animal models and patients with periodontal diseases, several specific oral bacteria associated with periodontitis can also alter the composition of the microbiota in the colon, thereby causing intestinal dysbiosis [4–7]. Some chronic diseases associated with periodontal disease have also been linked with gut dysbiosis [8]. The oral cavity could serve as a reservoir for intestinal pathobionts and contribute to the pathogenesis of diseases, including rheumatoid arthritis, atherosclerosis, liver cirrhosis, and colon cancer [9, 10]. In addition to infected periodontal tissues, recent studies have suggested that the intestine may serve as an important site of host immune modulation that enhances systemic inflammation via enhancements of multiple pro-inflammatory molecules involved in systemic inflammation and exacerbation of bone loss; include prostaglandin (PG)E_2_, interleukin (IL)-1β, and IL-6 [4, 5, 10, 11]. These findings of the prior studies suggest involvement of gut microbiota in the mechanisms by which periodontitis may induce and maintain a chronic state of inflammation at sites distant from the oral cavity.

There is considerable evidence to suggest that the gut microbiota modulates inflammatory processes and participates in the pathogenesis of T2DM and insulin resistance; it might also participate in treatments for affected patients. Specific alterations in gut microbiota could cause elderly individuals to become more susceptible to T2DM-related health problems [12, 13]. To the best of our knowledge, there has been little direct evidence has shown that this shift is driven by periodontitis. Although there have been some studies regarding gut microbial dysbiosis in elderly individuals with T2DM, these studies typically did not investigate the variables associated with periodontal status [12].

In this study, we investigated the complex influences of periodontitis on the development of T2DM in elderly individuals, including the underlying mechanisms associated with gut microbiota and systemic inflammation. Specifically, we used novel bioinformatics analysis combined with high-fidelity 16S rRNA gene amplicon sequencing technology to characterize the gut microbiota of patients with T2DM and periodontitis, compared with patients with T2DM alone and healthy volunteers in a parallel observational case-control study. We implemented a systematic approach to adjust for potential confounders. Furthermore, using the random forest algorithm, we identified key gut microbiota markers that distinguished participants with different periodontal conditions. Their relationships with various disease indexes, use of medications, and peripheral risk markers were analyzed by using Spearman correlation analysis. We expect that our findings will aid in systematic elucidation of the relationship between gut microbiota composition and inflammatory status in patients with periodontitis.

## RESULTS

### Gut dysbiosis in patients with T2DM and periodontitis

**Figure 1:**
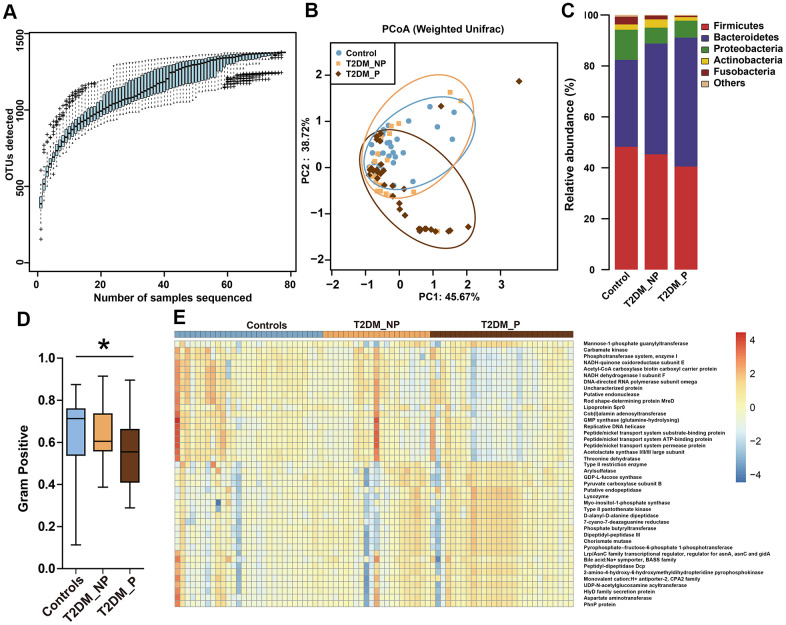
**Differences in gut microbiota profile among three groups.** (**A**) OTU accumulation boxplot to show that the number of samples is adequate. (**B**) Principal Co-ordinates Analysis (PCoA) plot of weighted UniFrac distances. (**C**) Fecal microbiota composition at the phylum level among the three groups. (**D**) BugBase algorithm predicted that microbiota phenotypes of the T2DM_P group significantly differed from healthy volunteers in terms of gram-positive bacteria. (**E**) Heatmap of PICRUSt analysis shows significant KEGG pathways among the three groups.

We analyzed fecal samples from 78 participants aged ≥ 65 years and evaluated the influence of periodontitis on gut microbiota of elderly patients with T2DM. The clinical characteristics and pathological indexes of participants are summarized in Table 1. There were no significant differences among groups in terms of age, sex, or BMI. All 78 DNA samples were analyzed for 16S rRNA gene amplification and products were sequenced. OTU accumulation boxplots were plotted to confirm that the sample size was adequate for comparative analysis (Figure 1A). The α-diversity indexes (Shannon and Simpson) did not significantly differ among the three groups (Supplementary Table 1). The environmental factors collected for analysis of β-diversity included basic clinical characteristics in this study. We focused on the major digestive tract complications that might exhibit associations with gut microbiota, including dry mouth and gastrointestinal (GI) symptoms (e.g., abdominal pain, abdominal bloating or distension, nausea/vomiting, gastroesophageal reflux, dysphagia, diarrhea, and constipation). Metadata were collected regarding 15 potential confounders, such as age, sex, BMI, duration of T2DM, hypertension, and GI symptoms, as well as use of medications (e.g., acarbose, metformin, aspirin, statins, calcium channel blockers, angiotensin receptor blockers, and pioglitazone). Eleven of the 17 variables exhibited collinearity (variance inflation factor > 2) and were therefore excluded (Supplementary Table 2). The number of remaining natural teeth, CPI score, and five other variables were used as environmental variables for PERMANOVA assessment. After adjustment for confounders, periodontal condition explained 6.6% of the variation in Bray–Curtis dissimilarity, 5.9% of the variation in unweighted UniFrac distance, and 2.3% of the variation in weighted UniFrac distance (Figure 1B, *P* < 0.05, PERMANOVA, Supplementary Table 3).

**Table 1:** Clinical characteristics of participants.

		**Controls**	**T2DM_NP**	**T2DM_ P**
		**(n = 29)**	**(n = 21)**	**(n = 28)**
Sex	Female	37.93% (11)	33.33% (7)	39.29% (11)
	Male	62.07% (18)	67.78% (14)	60.71% (17)
Age (year)		75.83±7.04	76.85±7.89	75.18±5.85
BMI		25.06±0.63	25.22±0.67	24.89±0.76
Duration of diabetes (year)		0	3.67±2.72	3.64±2.95***
*Hypertension* (%)		41.38% (12)	57.14% (12)	60.71% (17)
**Periodontal index**				
Number of teeth		25.10±2.43	24.38±4.22	14.82±1.59***^###^
Mean pocket depth (mm)		2.91±0.43	3.01±0.40	6.48±0.59***^###^
Bleeding on probing (%)		0% (0)	0% (0)	75% (21) ***^###^
Clinical attachment level (mm)		2.59±0.50	2.76±0.44	4.89±0.59***^###^
CPI		0.31±0.47	0.48±0.51	4.00±0***^###^
**Digestive tract symptoms**				
Dry mouth		55.17% (16)	76.19% (16)	75% (21)
GI symptoms		34.28±3.07	41.95±9.90	41.00±9.27*
**Medicine and therapeutic interventions**				
Insulin treatment (%)		0% (0)	19.05% (4)	17.86% (5) *
Hypogylcemic agents (%)	Acarbose	0% (0)	23.81% (5)	21.43% (6) *
	Pioglitazone	0% (0)	14.29% (3)	11.00% (3)
	Metformin	0% (0)	9.52% (2)	7.14% (2)
Hypotensive agents (%)	CCBs	27.59% (8)	38.10% (8)	50.00% (14)
	ARBs	13.79% (4)	19.05% (4)	39.29% (11) *
Cholesterol-lowering drugs (%)	Statins	13.79% (4)	19.05% (4)	17.86% (5)
Antithrombotic agents (%)	Aspirin	6.90% (4)	42.86% (9)	28.57% (8)

Abbreviations: T2DM_P: patients with type 2 diabetes mellitus and periodontitis; T2DM_NP: patients with type 2 diabetes mellitus alone (no periodontitis); BMI: body mass index; CPI: Community Periodontal Index; GI: gastrointestinal; CCBs: calcium channel blockers; ARBs: angiotensin receptor blockers.

Continuous variables are presented as mean ± SD. Significant differences between T2DM_NP and T2DM_P groups are indicated by *#P* < 0.05, *## P* < 0.01, and *### P* < 0.001. Significant differences between healthy volunteers and both groups of patients with T2DM are indicated by *P < 0.05, **P < 0.01, and ***P < 0.001.

The dominant phyla among participants were *Firmicutes, Bacteroidetes*, and *Proteobacteria* (Figure 1C). BugBase algorithm-based prediction suggested that the T2DM_P group exhibited preferential enrichment of gram-positive taxa, compared with the healthy volunteers (Figure 1D). Predicted microbiota functions were determined by KEGG pathway analysis (Figure 1E, Supplementary Table 4). Pathways associated with cytotoxicity that were predicted to be upregulated in the T2DM_P group included the HlyD family secretion protein, UDP-N-acetylglucosamine acyltransferase, and chorismate mutase (Kruskal-Wallis test *P* < 0.05). Pathways predicted to be downregulated in the T2DM_P group included carbamate kinase, threonine dehydratase, NADH-quinone oxidoreductase subunit E, and acetolactate synthase I/II/III large subunit (Kruskal-Wallis test *P* < 0.05).

### Disturbance in gut flora interaction driven by periodontitis

**Figure 2:**
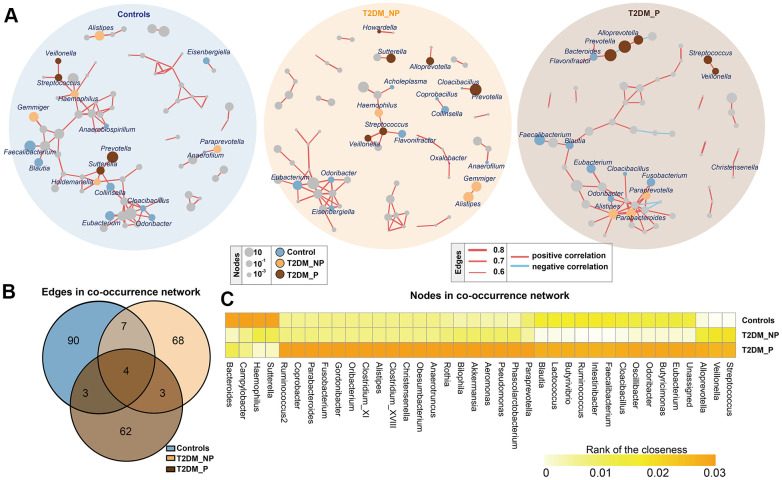
**Co-occurrence networks of gut microbiota in different groups.** (**A**) Co-occurrence network analysis of bacterial genera with correlation coefficient >0.6 or <-0.6 in each group. Each network node indicates a bacterial genus. Circle size increases with relative abundance. Circle colors correspond to biomarkers characteristic of the groups in this study; other bacterial genera are shown in gray. Edge widths represents correlation values supporting this connection. Edge colors show positive (red) and negative (blue) correlations, respectively. (**B**) Numbers of unique and shared edges in three co-occurrence networks. (**C**) Centralities (rank of closeness) and discrepancies of nodes in different groups.

To infer potential interactions among gut bacterial community members, we constructed co-occurrence networks of bacterial genera, based on correlations of their relative abundances (Figure 2). Overall, the co-occurrence network of the T2DM_P group exhibited greater complexity with distinct components and topographies, compared with other groups (Figure 2A). The co-occurrence network of the T2DM_P group was characterized by two unique modules separate from the core module (one comprised *Prevotella, Bacteroides,* and *Alloprevotella*, and the other comprised *Streptococcus* and *Veillonella*). Specifically, the co-occurrence networks of the T2DM_P and T2DM_NP groups contained 72 and 82 edges, respectively; the co-occurrence network of the control group contained 104 edges (Figure 2B). Despite the presence of some overlapping edges, many edges were specific to each group. Notably, correlations between *Romboutsia* and *Turicibacter*, and between *Streptococcus* and *Veillonella*, were present in all three groups. Connections containing *Butyricimonas*, *Odoribacter*, *Oscillibacter*, and *Eubacterium* showed high frequencies of co-occurrence in samples from patients with T2DM, suggesting they were universally associated with T2DM. In addition, the T2DM_P group had more co-exclusion relationships in the bacterial community; in particular, antagonistic relationships between *Alloprevotella* and *Methanobrevibacter*, as well as between *Bilophila* and *Actinoplanes*, were only observed in the T2DM_P group. We then computed different bacterial network characteristics (i.e., degree, closeness, and betweenness) of shared nodes among bacterial networks to approximate the structural importance of individual bacterial taxa within each respective network (Figure 2C). In terms of the closeness centrality of shared nodes among bacterial networks, several genera appeared to have important effects in the T2DM_P network, including *Alistipes, Bilophila, Butyricimonas, Faecalibacterium,* *Parabacteroides, Streptococcus, Veillonella, Odoribacter*, and *Pseudomonas*.

### Identification of key gut microbiota taxa associated with periodontitis by random forest and LEfSe

**Figure 3:**
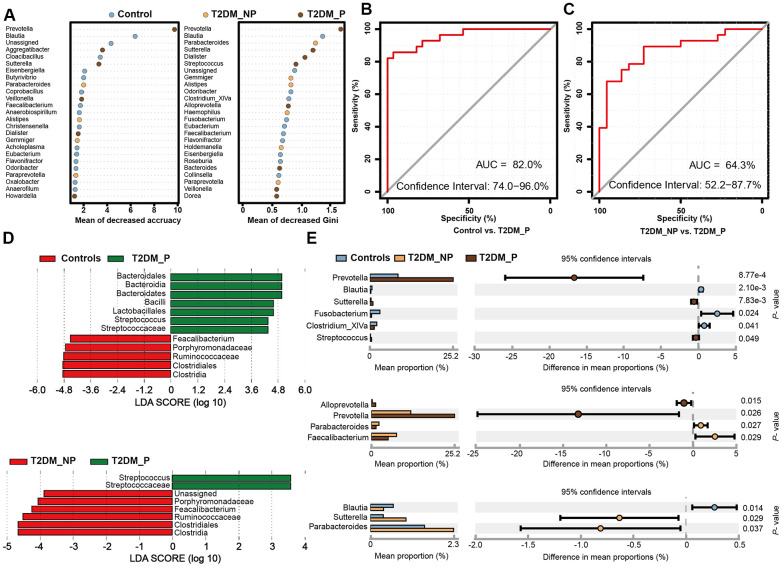
**Microbial biomarkers between healthy volunteers and patients with diabetes and periodontitis or diabetes alone.** (**A**) Microbial markers of the T2DM_P group were identified with random forest models. The 34 OTU markers were selected by random forest models as optimal markers to distinguish among groups. (**B, C**) AUCs were 85.84% (95% CI, 76.3%-95.3%) between T2DM_P and control cohorts (**B**), and 78.83% (95% CI, 65.4%-92.3%) between T2DM_P and T2DM_NP cohorts (**C**). (**D**) Linear discriminant analysis (LDA) revealed differentially abundant taxa as biomarkers using the Kruskal-Wallis test (P < 0.05) with LDA score > 2.0. (**E**) The Wilcoxon rank-sum test was used for comparisons of relative abundances at the genus level in different groups.

To identify key gut microbiota markers that distinguished participants with different periodontal conditions, we performed fivefold cross-validation analysis using a random forest model (Figure 3A). We created a ROC curve to measure the accuracies at which the relative abundances of key gut microbiota related to periodontitis were able to classify two groups of samples (Figure 3B, 3C). The top 34 OTU markers identified by random forest analysis were able to distinguish the T2DM_P group from healthy volunteers with an area under the ROC curve of 0.82 (95% confidence interval: 74.0%–96.0%); they could distinguish the T2DM_P group from the T2DM_NP group with an area under the ROC of 0.64 (95% confidence interval: 52.2%–87.7%). In addition, we performed LEfSe analysis to confirm the composition of significant differences between groups at various taxonomic levels, especially the *Faecalibacterium* genus, for which significance was confirmed by the Wilcoxon rank-sum test (Figure 3D, 3E). However, because of high inter-individual variabilities, only seven taxa exhibited significant differences in relative abundance between groups after *FDR* adjustment (Kruskal-Wallis test, adjusted for age, sex, and use of acarbose; *FDR*-corrected *P* < 0.05, Supplementary Table 5). Overall, we found that the abundance of the genus *Prevotella* was significantly elevated in the T2DM_P group, while the abundance of the genus *Faecalibacterium* was significantly depleted. At the species level, significantly altered abundances of *Prevotella copri* and *Faecalibacterium prausnitzii* (the only species thus far defined in the *Faecalibacterium* genus) were observed in the T2DM_P group.

### Clinical correlates of key gut microbiota markers associated with periodontitis

**Figure 4:**
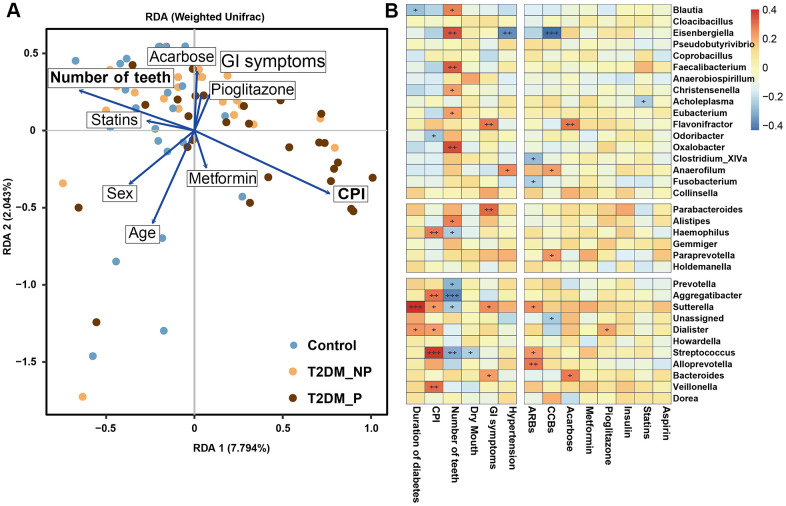
**The clinical correlates of gut microbiota at genus level.** (**A**) Redundancy analysis (RDA) plot of bacterial diversity and seven non-collinear variables. (**B**) Correlation between the gut microbial biomarkers and multiple clinical parameters.

Clinical factors associated with T2DM might shape the gut microbiota through long-term effects on the ecological balance between the host and the normal microbiota, including GI symptoms and a variety of medications that are used to treat T2DM. We performed forward selection analysis and redundancy analysis to evaluate the relationships between these features and the relative abundances of gut microbiota at the genus level (Figure 4A). The results indicated that the number of teeth, CPI, age, sex, and acarbose use exhibited greater power as driving factors; metformin use, statin use, pioglitazone use, and abdominal discomfort exhibited weaker impact. Only the number of teeth and CPI had significant effects (*P* = 0.003 and *P* = 0.02, respectively, Supplementary Table 6). Subsequently, we explored the correlation between the key gut microbiota markers identified above and clinical characteristics, including various disease indexes and use of medications (Figure 4B, Supplementary Tables 7, 8). The duration of diabetes was positively correlated with the relative abundances of *Sutterella* and *Dialister* and negatively correlated with the relative abundance of *Blautia.* The CPI score was positively correlated with the relative abundances of *Haemophilus*, *Veillonella,* *Streptococcus*, and *Aggregatibacter*; the number of teeth was positively correlated with the relative abundances of *Faecalibacterium, Oxalobacter,* and *Eisenbergiella* and negatively correlated with the relative abundances of *Prevotella, Aggregatibacter,* and *Streptococcus.* Dry mouth, another oral complication of T2DM linked to periodontitis, was positively correlated with the relative abundance of *Dialister* and negatively correlated with the relative abundance of *Coprobacillus*. Furthermore, we found that GI symptoms was positively correlated with the relative abundances of *Flavonifractor, Parabacteroides,* *Sutterella*, and *Bacteroides*. Notably, the relative abundances of *Flavonifractor* and *Bacteroides* were also positively correlated with acarbose use. In addition, the relative abundance of *Eisenbergiella* was significantly negatively correlated with hypertension and calcium channel blocker use.

**Figure 5:**
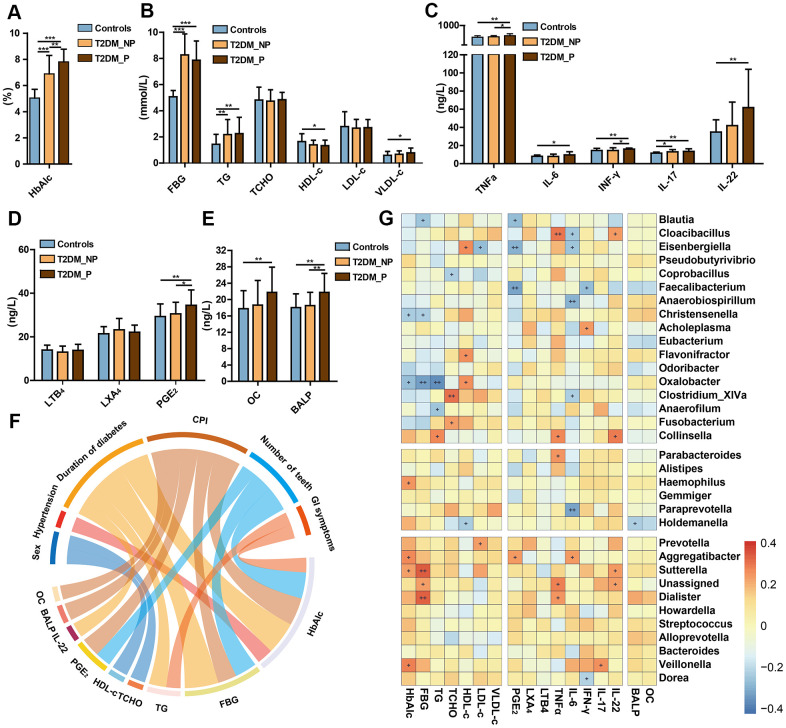
**Relationships of periodontal condition, altered gut microbiota, and peripheral risk markers in elderly individuals.** Levels of HbA1c (**A**), FBG and lipids (**B**), cytokines (**C**), eicosanoids (**D**), and bone metabolic markers (**E**) in T2DM_P, T2DM_NP, and healthy volunteer groups (shown as mean +/- SD). Statistics were calculated by Student's t-test. Significant differences between groups are indicated by *P < 0.05, **P < 0.01, and ***P < 0.001. (**F**) Circos plot shows significant correlation values for comparisons between plasma risk factors and clinical characteristics in all participants with correlation coefficient > 0.3 or < -0.3. Colors correspond to the direction of correlation; warm shades denote positive relationships and cool tones denote negative correlations. (**G**) Heatmap depicts correlations between gut microbial biomarkers and peripheral risk markers. Non-parametric Spearman correlation coefficients were calculated between each of 34 gut microbial biomarkers and peripheral risk markers. Colors range from blue (negative correlation) to red (positive correlation). Significant relationships are indicated by +P < 0.05, ++P < 0.01, and +++P < 0.001.

We also investigated the influences of periodontitis on metabolic and inflammatory profiles. The abundances of periodontitis-associated gut microbiota taxa (e.g., *Prevotella, Faecalibacterium, Haemophilus*, *Veillonella,* *Streptococcus*, *Aggregatibacter*, *Oxalobacter,* and *Eisenbergiella*) also exhibited significant correlations with the blood levels of pro-inflammatory cytokines (e.g., PGE_2_, IFN-γ, IL-17, IL-6, and TNF-α) and plasma metabolic parameters (e.g., HbA1c, fasting blood glucose [FBG], total cholesterol [TCHO], high-density lipoprotein cholesterol [HDL-c], and low-density lipoprotein cholesterol [LDL-c]). Specifically, the T2DM_P group exhibited a significantly elevated Hemoglobin A1c (HbA1c) level, compared with the T2DM_NP group (*P* = 0.009, Figure 5A); there were no significant differences in other serum metabolic parameters between the T2DM_P and T2DM_NP groups (Figure 5B). Adverse systemic inflammation was observed in the T2DM_P group, compared with the T2DM_NP group. Several inflammatory factors were significantly elevated in the T2DM_P group, including serum levels of tumor necrosis factor α (TNF-α) and Prostaglandin E2 (PGE_2_) (*P* = 0.030 and *P* = 0.036, respectively; Figure 5C and 5D). In addition, bone loss was more serious in the T2DM_P group, as indicated by higher levels of bone alkaline phosphatase and osteocalcin (Figure 5E). Circos plot analysis revealed correlations between clinical characteristics and peripheral risk factors. Correlations were observed between CPI and peripheral concentrations of both PGE_2_ and HbA1c; a significant correlation was also observed between IL-22 and duration of diabetes. Figure 5G shows a heatmap of the correlation coefficients between circulating metabolic markers and key gut microbiota taxa, as well as between circulating inflammatory markers and key gut microbiota taxa (Supplementary Table 9, 10). The relative abundance of *Faecalibacterium* was significantly negatively correlated with serum levels of PGE_2_ and interferon-γ (IFN-γ), whereas the relative abundance of *Prevotella* was significantly positively correlated with plasma low-density lipoprotein level. Serum levels of PGE_2_ and IL-6 were negatively correlated with relative abundance of *Eisenbergiella* and positively correlated with the relative abundance of *Aggregatibacter*. Additionally, a positive association was observed between the relative abundance of *Veillonella* and IL-17, while a negative association was observed between the relative abundance of *Dorea* and IFN-γ; these findings suggested probable contributions to systemic inflammatory responses in periodontitis.

## DISCUSSION

In the present study, we identified key characteristics of gut microbial dysbiosis driven by periodontal complications among elderly patients with T2DM. The results of PERMANOVA community and redundancy analysis indicated that the number of remaining natural teeth and CPI score were the main factors that drove the separation in gut microbiota. Their reversible effects on gut microbiota were more pronounced than the known effects of other influential factors including age, sex and acarbose use [14]. We consistently observed unfavorable metabolic and inflammatory profiles among patients in the T2DM_P group, compared with healthy volunteers or patients in the T2DM_NP group. Our findings showed that altered gut microbiota in patients with T2DM were linked to their periodontal conditions and correlated with several risk factors for advanced T2DM and bone loss.

Here, we identified the 34 most-discriminatory genera associated with periodontitis by fivefold cross-validation using a random forest model and LEfSe. Of the 34 genera, 13 included species that are natural members of the human oral biofilm community (e.g., *Streptococcus*, *Fusobacterium, Veillonella, Dialister, Haemophilus, Aggregatibacter, Prevotella, Gemmiger, Cloacibacillus, Odoribacter, Howardella, Eubacterium*, and *Bacteroides*) [15–17]. The oral bacteria swallowed with food and saliva generally interact with gut microbiota. Given the competitive and cooperative relationships among microbial groups, the effects of periodontitis could presumably span across distant communities along the digestive tract. Here, we found that the most striking differences in co-occurrence networks between the T2DM_P and T2DM_NP groups comprised interactions involving dominant genera in the oral cavity (i.e., *Streptococcus* and *Veillonella*); sequences of DNA from these bacteria have also been identified in atherosclerotic plaques [8]. Our findings are consistent with the findings of recent studies, in which oral bacteria were shown to travel into the colon [10]. In addition to periodontal pathogens, other members of the oral microbiota are reportedly involved in the development of systemic diseases associated with intestinal dysbiosis.

In this study, we found that the relative abundance of *P. copri* was significantly enriched in the T2DM_P group after adjustments for age, sex, and acarbose use, compared with the other two groups. Prior studies in humans and animals have shown strong relationships between *P. copri* and chronic inflammatory diseases, such as rheumatoid arthritis and colitis [4, 18–20]. The significantly elevated abundance of *P. copri* in the T2DM_P group is consistent with previously reported findings in Brazilian patients with T2DM [19]. In contrast, patients in the T2DM_P group showed a more pronounced reduction of the abundance of the anti-inflammatory species *F. prausnitzii*, compared with both healthy controls and patients in the T2DM_NP group. *F. prausnitzii* has been shown to enhance insulin sensitivity and inhibit gastric emptying by stimulation of GLP-1 secretion [4]. Previous experimental and clinical studies have suggested the possible involvement of the beneficial butyrate-producing bacteria *F. prausnitzii* in the pathogeneses of both T2DM and frailty [13, 21, 22].

Recent data suggested a plausible mechanism for systemic chronic inflammatory status and insulin resistance involving microbial dysbiosis, with resulting modulation of the innate immune response to endotoxin challenge [23]. Notably, the predicted microbiota functions determined by KEGG pathway analysis indicated that a range of pathways associated with cytotoxicity were highly likely to be induced in the T2DM_P group. These pathways include the HlyD family secretion protein pathway, which is presumed to play a role in the secretion of substrates (e.g., cytotoxin VacA from *Helicobacter pylori* and pertactin from *Bordetella pertussis*), and the UDP-N-acetylglucosamine acyltransferase pathway, which is associated with endotoxin biosynthesis (e.g., lipid A and lipopolysaccharide) [24]*.* These observations support a potential mechanism linking periodontitis and gut dysbiosis in which chronic oral ingestion of high doses of periodontopathic microorganisms (including dead bacteria from the oral cavity) may stimulate several pathogens in the gut and upregulate bacterial virulence genes; this chronic stimulation and upregulation may lead to endotoxemia and subsequent metabolic disorders [8]. The continuous infusion of lipopolysaccharide from *Porphyromonas gingivalis* and associated periodontal dysbiosis has been shown to induce a chronic low-grade systemic inflammation leading to the insulin resistance in mice fed a high-fat diet [25]. Furthermore, a clinical study demonstrated the potential for oral hygiene control habits to improve glycemic control and oral malodor in patients with T2DM [26].

Consistent with this hypothesis, the present study showed that circulating levels of pro-inflammatory mediators (i.e., PGE_2_ and TNF-α) were higher in the T2DM_P group than in the T2DM_NP group in this study. Of particular interest was PGE_2_, which reportedly exhibited significant upregulation in gingival tissue and gingival fluid from patients with periodontitis [27]. In this study, we found that the serum level of PGE_2_ was positively correlated with the relative abundance of *Aggregatibacter* and negatively correlated with the relative abundances of *Blautia, Eisenbergiella*, and *Faecalibacterium*. Notably, elevated production of PGE_2_, TNF-α, and IL-6, following stimulation with lipopolysaccharide from *Aggregatibacter actinomycetemcomitans*, has been observed in IL-1 receptor antagonist-deficient mice [28].

Previous studies in animal models and humans have identified specific alterations in gut microbiota community structure in association with the presence of T2DM [30]. However, most studies have regarded T2DM as a single predictor and have neglected a wide range of variables and complications in elderly patients that might influence gut microbiota. In this study, we focused on periodontal conditions and utilized a systematic approach to control for potential confounders, including age, sex, comorbidities, and use of medications. The results highlight a need to consider periodontal status as a potential confounding factor in studies linking gut microbiota differences to T2DM. Significant differences at the genus level were difficult to detect because many genera exhibited very low abundances; moreover, there were considerable interindividual differences in gut microbiota composition among elderly individuals, particularly in patients with T2DM whose exhibited complex clinical conditions. Thus, in addition to widely used approaches for the identification of taxonomic differences among groups, we performed fivefold cross-validation using a random forest model to identify key gut microbiota taxa that could distinguish patients according to periodontal status. Our systematic approach is likely to be useful for other researchers who are investigating variations in the gut microbiota during aging and their associations with disease. The results of this study highlight a need to consider periodontal status as a potential confounding factor in studies linking gut microbiota differences to T2DM.

Talita et al. [4] also investigated the relationship between periodontitis and gut dysbiosis in a parallel observational case-control study. They found that individuals with periodontal diseases exhibited a less diverse gut microbiome, characterized by an enhanced Firmicutes/Bacteroidetes ratio. This finding differs from our results, presumably because their analysis was based on a young population, excluding patients with history of metabolic disease or abnormal gastrointestinal symptoms was excluded. Notably, the Prevotella genus showed significant associations with periodontal disease in both young and older adults. The association of the Prevotella genus with diabetes has been observed across generations and body sites [18]. Some species of Prevotella exhibit distinct pathobiontic properties [29]. Our data also showed that most identified OTUs belong to *Prevotella copri*. Taken all together, our data support the hypothesis that gut microbial shifts driven by periodontitis contribute to systemic inflammation and metabolic dysfunction which may in turn accelerate the progression of T2DM. The underlying mechanism behind this association deserves further attention through experimental modeling and larger longitudinal clinical investigations.

The limitations of the current study were as follows. First, limited long-term diet information was available for the elderly patients in this study; thus, individual nutritional statuses were not considered. Second, our finding that periodontitis was related to gut bacterial composition must be interpreted with caution, because our dataset was limited by considerable interindividual variability in gut microbiota among participants. However, by using various bioinformatics algorithms, we were able to detect signature bacteria with discriminative importance that persisted after rigorous statistical testing and adjustment. Further analyses are required to yield further insights regarding the mechanism by which the combined effects of microbe–microbe and host–microbe interactions drive gut microbiota differentiation in elderly patients with chronic oral infections.

In conclusion, our findings are expected to aid in systemic elucidation of the relationship between gut microbiota composition and inflammatory status in patients with periodontitis, and in identifying the probable roles of these periodontitis-associated gut bacteria in T2DM pathogenesis. Our observations extend the current knowledge regarding periodontitis-related alterations in gut microbiota of elderly patients with T2DM.

## MATERIALS AND METHODS

### Study design and participants

Ethics approval for the study was obtained from the ethics board of the First Affiliated Hospital, School of Medicine, Zhejiang University (IRB no. 2014-114). Written informed consent and questionnaire responses were obtained from all participants in this study. Patients with T2DM, with or without periodontitis, were enrolled in the study by their physicians. Patients provided fecal and blood samples if they fulfilled the following criteria: (1) age ≥ 65 years with body mass index (BMI) of 17–38; (2) no antibiotic use in the 12 weeks prior to enrollment. Patients were included in the T2DM_P group if they exhibited periodontitis (e.g., chronic periodontitis, aggressive periodontitis, gingivitis, periodontal abscess, or combined periodontic-endodontic lesions). Patients were included in the T2DM_NP group if they did not exhibit periodontitis. The exclusion criteria for this study were: (1) presence of acute illness (e.g., upper tract respiratory infections, pneumonitis, or enterocolitis); (2) presence of any other organ-specific diseases (e.g., intestinal diseases, pancreatic diseases, tumor recurrence, heart failure, renal failure, stroke, and/or peripheral artery disease); (3) presence of severe T2DM complications (e.g., stroke, retinopathy, and nephropathy); and (4) consumption of alcohol, tobacco, Chinese herbal medicine, and/or recreational drugs. We reviewed the medical records of 49 patients aged ≥ 65 years with T2DM (28 with periodontitis and 21 without periodontitis) and the records of 29 age- and sex-matched healthy volunteers from the same community. All healthy volunteers were within the normal ranges upon physical examination; had no history of smoking or alcohol abuse; had no airway infection, runny nose, sputum production, or other systemic disease; and had not received antibiotics, probiotics, or prebiotics in the month before enrollment. At each clinic visit, participants described their use of medications, including acarbose, metformin, aspirin, statins, calcium channel blockers, angiotensin receptor blockers, and pioglitazone.

Periodontal evaluation was performed promptly by trained dentists and the severity of periodontitis was assessed by simple World Health Organization Community Periodontal Index (CPI) codes [30]. A detailed medical and dental history was taken for each participant, followed by an oral examination that included a periodontal examination. The CPI was scored on a scale of 0 to 4, as follows: 0 = healthy periodontal tissue (no bleeding, calculus, or pocket depth ≥ 3.5 mm); 1 = bleeding in periodontal tissue (bleeding on probing, but no calculus or pocket depth ≥ 3.5 mm); 2 = supragingival or subgingival calculus; 3 = 3.5–5.5-mm-deep periodontal pockets; and 4 = ≥ 5.5-mm-deep periodontal pockets.

The Diabetes Bowel Symptom Questionnaire was used to assess the severity of GI symptoms (Supplementary Table 11). Details of the questionnaire have been described previously [31]. The questionnaire consisted of parts A and B, which contained detailed information regarding seven symptoms/symptom complexes occurring within the prior 3 months: abdominal pain; abdominal bloating or distension; nausea/vomiting; gastroesophageal reflux; dysphagia; diarrhea; and constipation. Thirty-two questions were designed to examine the presence and severity of abdominal pain/discomfort (Part A) and upper GI symptoms (Part B). Higher scores indicated higher frequency or severity of symptoms.

### Laboratory evaluations of plasma biochemical profile and inflammatory markers

Venous blood samples were taken for measurement of standard hematological and clinical chemistry variables. Plasma biochemical profiles were assessed in all participants using standard in-hospital methods, including HbA1c, FBG, TG, TCHO, HDL-c, LDL-c, VLDL-c, BALP, and OC. The plasma concentrations of TNF-α, IL-6, IFN-γ, IL-17, IL-22, IL-25, and PGE_2_ were measured by enzyme-linked immunosorbent assay kits (4A Biotech Co., Beijing, China). All assays were conducted in duplicate and in accordance with the manufacturer’s instructions. Biochemical indexes were measured by the hospital central diagnostic laboratory.

### 16S rRNA gene tag sequencing

Fecal samples (250 mg, wet weight) were immediately stored at −80° C until DNA extraction. The protocols for fecal bacterial DNA extraction, V3–V4 amplification, and sequencing were performed as described in our previous study [32]. Briefly, total fecal bacterial DNA was isolated using the Qiagen Mini Kit (Qiagen, Hilden, Germany), and molecular sizes were estimated by means of agarose gel electrophoresis. V3 and V4 regions were amplified by polymerase chain reaction using the following primer pair: 338F, 5’-barcode-ACTCCTACGGGAGGCAGCA-3’ and 806R, 5’-GGACTACHVGGGTWTCTAAT-3’. Polymerase chain reaction amplification was then performed as described in our previous study [33]. DNA libraries were constructed using kits provided by Illumina Inc. (San Diego, CA, USA), in accordance with the manufacturer’s instructions; DNA sequencing was performed using the Illumina MiSeq 2000 platform. Sequence data have been deposited under NCBI BioProject accession number PRJNA655208.

Quality-filtered sequences were clustered into unique sequences and sorted in order of decreasing abundance to identify representative sequences using UPARSE [34]. Sequences were grouped into operational taxonomic units (OTUs) using the clustering program VSEARCH [35]. The phylogenetic affiliation of each 16S rRNA gene sequence was analyzed using RDP Classifier (http://rdp.cme.msu.edu/) against the Silva (SSU132) 16S rRNA database, with a confidence threshold of 70%. 16S rDNA V3-V4 region amplicon sequencing generated 5,154,465 high-quality reads, with an average of 69,915 reads (range: 52,536–89,360) per sample.

### Bioinformatics and statistical analysis

OTUs were grouped at different levels of taxonomy classification (from phylum to species) following the Bayesian approach with a 97% cutoff value. OTU-based α-diversity was estimated by calculating the following indexes: Chao1, observed OTUs, Simpson, and Shannon. Differences in these indexes among the three groups were analyzed by using Kruskal–Wallis tests. β-diversity was estimated by computing the Bray–Curtis dissimilarity, weighted UniFrac distance, and unweighted UniFrac distance; it was visualized using principal coordinate analysis, and the results were plotted using the WGCNA, stats, and ggplot2 packages in R software (Version 3.4.4, http://www.R-project.org/). To probe the microbial metabolism and predict metagenome functional content from marker genes, PICRUSt analysis was performed to explore differences in KEGG pathways among groups [36].

The variables were tested for collinearity with the CPI using variance inflation factor in the HH package in R. To ensure that the choice of the metric did not affect the results, the distances were calculated using three metrics: unweighted UniFrac distance, weighted UniFrac distance, and Bray–Curtis dissimilarity. Constrained ordination redundancy analysis was implemented in the vegan package in R, in accordance with previously described methods [37]. The significance of environmental variables was checked by forward selection using the ordistep function in the vegan package, with 99,999 permutations. A rarefied OTU table was used for all three metrics. UniFrac distances were calculated in QIIME 1.9.1 and Bray–Curtis dissimilarity was calculated in the vegan package in R. Permutational MANOVA (PERMANOVA) was performed on the UniFrac distances and Bray–Curtis dissimilarity using the adonis function in the vegan package in R, with 99,999 permutations. To test for confounding, adjusted PERMANOVA was conducted with T2DM_P and seven covariates in the model; the marginal effects were tested in accordance with previously described methods [38].

adonis(dis~P_status + Age + Sex + Abdominal_discomfort + Acarbose + Pioglitazone + Metformin + Statin, group, permutations = 99999)

Differences were tested at species, genus, family, order, class, and phylum levels. Taxa present in <10% of samples were removed; thus, 83 species, 113 genera, 45 families, 30 orders, 20 classes, and 12 phyla were included. Taxon abundances were compared between T2DM_P and controls, as well as between T2DM_P and T2DM_NP. Linear discriminant analysis effect size (LEfSe, http://huttenhower.sph.harvard.edu/lefse/) was used to characterize microorganismal features that distinguished gut microbiota specific to the T2DM_P group at multiple taxonomic levels, grade biomarkers according to statistical significance, and visualize the results in bar charts. Community analysis and differential abundance of OTUs were performed using the Statistical Analysis of Metagenomic Profiles software. Both analyses incorporated false-discovery rate (*FDR*) correction for multiple testing (*FDR*<0.05). To test for potential confounding, significant taxa were retested with adjustment for covariates, using a generalized linear model with negative binomial distribution and controlling for zero-inflation as appropriate, in the glmmADMB package in R:

Taxon~P-status Age+ Sex + Acarbose use

As previously described [39], identification of signature bacteria was performed by fivefold cross-validation analysis on a random forest model (R 3.4.1, randomForest 4.6–12 package) and receiver operating characteristic (ROC) curves were constructed using the pROC package.

The co-occurrence networks of gut microbiota were visualized using the igraph package in R. Closeness and eigenvector of the nodes were calculated to measure node centralities in each network, as described previously [18]. Genera with relative abundance > 0.05% were subjected to Spearman correlation analyses of their occurrence patterns. The bacterial correlations in samples from each group were computed based on the respective abundances of individual genera. Only strong correlations (*P* < 0.05 after *FDR* correction, correlation coefficient > 0.6) were visualized in this analysis. The microbiota networks of these three groups were compared; the numbers of edges were counted and centralities of nodes were determined in each co-bacterial occurrence network to quantify these differences.

Categorical variables were analyzed using the Chi-square or Fisher’s exact test. Continuous variables were compared using the Wilcoxon rank-sum test. Categorical variables were compared using Fisher’s exact test. The correlations of signature bacteria (identified by the random forest algorithm) with various clinical parameters (e.g., combined disease, metabolic profile, and inflammatory profile) were assessed using Spearman correlation analysis in R. Correlations were identified by Spearman’s rank correlation coefficient (significance thresholds were *P* < 0.05). Statistical analyses were performed using IBM SPSS Statistics for Windows V.20.0 (IBM Corp., Armonk, NY, USA). GraphPad Prism V.6.0 (GraphPad Software Inc., San Diego, CA, USA) and R were used for various analyses and preparation of graphs.

## Supplementary Material

Supplementary Tables
