# Supplementary material for: Periodontitis in elderly patients with type 2 diabetes mellitus: impact on gut microbiota and systemic inflammation
Source: Aging (Albany NY). 2020 Nov 25;12(24):25956–80. doi: 10.18632/aging.202174 (PMC7803515; doi:10.18632/aging.202174)
Supplement: Supplementary Tables [file aging-12-202174-s001.pdf]

SUPPLEMENTARY TABLES

Supplementary Table 1. α-Diversity indexes of gut microbiota among participants.

	Controls (n = 29)	T2DM_ NP (n = 21)	T2DM_ P (n = 28)	*P*-value Controls (n=29) vs T2DM_NP (n=21)	*P*-value Controls (n=29) vs T2DM_P (n=28)	*P*-value T2DM_NP (n=21) vs T2DM_P (n=28)	*P*-value Controls (n=29) vs T2DM_NP (n=21) vs T2DM_P (n=28)
**Observed OTUs**	397.45±164.07	381.29±136.96	402.64±75.82	0.83	0.69	0.28	0.64
**Chao1**	482.82±162.87	472.42±136.60	504.50±75.58	0.94	0.25	0.15	0.63
**Shannon**	3.73±1.08	3.76±0.75	3.57±0.75	0.88	0.33	0.35	0.89
**Simpson**	0.08±0.07	0.06±0.03	0.09±0.07	0.55	0.36	0.14	0.70

Abbreviations: T2DM_P: patients with type 2 diabetes mellitus and periodontitis; T2DM_NP: patients with type 2 diabetes mellitus alone (no periodontitis); OTUs: operational taxonomic units; ACE: abundance-based coverage estimator.
P-values were calculated with Kruskal–Wallis tests.

Supplementary Table 2. Variance inflation factor analyses of variables associated with microbiota (Figure1B).

**Variables**	**VIF**
Number of teeth	5.03
CPI	4.92
T2DM status	3.09
Age	1.48
Sex	1.27
BMI	1.63
Dry mouth	2.59
Duration of diabetes	12.89
Hypertension	9.30
GI symptoms	2.15
Acarbose	1.48
Metformin	1.61
Aspirin	2.16
Statins	1.66
Calcium channel blockers	7.27
Angiotensin receptor blockers	2.71
Pioglitazone	1.73

Abbreviations: CPI: Community Periodontal Index; T2DM: type 2 diabetes mellitus; GI: gastrointestinal.

Supplementary Table 3. Permutational multivariate analysis of variance, with or without confounder adjustment (Figure1B).

	Bray-curtis	Unweighted Unifrac	Weighted Unifrac
**(A) without confounder adjustment**			
Controls (n = 29) vs. T2DM_NP (n = 21) vs. T2DM_P (n = 28)	4E-3	4E-3	0.03
**(B) All subjects**			
Controls (n = 29) vs. T2DM_NP (n = 21) vs. T2DM_P (n = 28)	4E-3	4E-3	0.03
Age	0.54	0.29	0.10
Sex	0.93	0.87	0.29
GI symptoms	0.20	0.19	0.25
Acarbose	0.01	0.01	0.61
Pioglitazone	0.97	0.99	0.93
Metformin	0.76	0.84	0.66
Statins	0.45	0.58	0.37
**(C) Within T2DM subjects**			
T2DM_NP (n = 21) vs. T2DM_P (n = 28)	6E-3	0.01	6E-4
Age	0.54	0.51	0.07
Sex	0.56	0.50	0.33
GI symptoms	0.17	0.13	0.77
Acarbose	0.05	0.04	0.03
Pioglitazone	0.91	0.96	0.93
Metformin	0.60	0.66	0.39
Statins	0.55	0.76	0.11

Abbreviations: T2DM_P: patients with type 2 diabetes mellitus and periodontitis; T2DM_NP: patients with type 2 diabetes mellitus alone (no periodontitis).

Supplementary Table 4. Details on PICRUSt prediction (Figure 1E).

KEGG_Description	KEGG	Eta-squared	*P*-value	Corrected *P*-value
NADH dehydrogenase I subunit F [EC:1.6.5.3]; NADH-quinone oxidoreductase subunit F [EC:1.6.5.3]	g4033	0.278	1.20E-06	9.97E-05
acetyl-CoA carboxylase biotin carboxyl carrier protein	g6191	0.259	5.10E-06	2.12E-04
NADH-quinone oxidoreductase subunit E [EC:1.6.5.3]; NADH dehydrogenase I subunit E [EC:1.6.5.3]	g4034	0.281	1.10E-05	3.03E-04
DNA-directed RNA polymerase subunit omega [EC:2.7.7.6]	g1676	0.193	2.39E-05	4.96E-04
threonine dehydratase [EC:4.3.1.19]	g2801	0.251	2.86E-05	4.75E-04
peptide/nickel transport system ATP-binding protein	g5254	0.144	3.51E-05	4.85E-04
acetolactate synthase I/II/III large subunit [EC:2.2.1.6]	g3654	0.232	4.09E-05	4.85E-04
putative endonuclease	g1179	0.163	5.13E-05	5.33E-04
cob(I)alamin adenosyltransferase [EC:2.5.1.17]	g5815	0.249	5.25E-05	4.84E-04
peptide/nickel transport system substrate-binding protein	g5257	0.164	5.68E-05	4.71E-04
peptide/nickel transport system permease protein	g5255	0.151	9.13E-05	6.89E-04
myo-inositol-1-phosphate synthase [EC:5.5.1.4]	g4460	0.191	1.13E-04	7.83E-04
pyrophosphate--fructose-6-phosphate 1-phosphotransferase [EC:2.7.1.90]	g1538	0.211	1.15E-04	7.36E-04
rod shape-determining protein MreD	g4647	0.131	1.88E-04	1.12E-03
dipeptidyl-peptidase III [EC:3.4.14.4]	g2471	0.18	2.53E-04	1.40E-03
dipeptidyl-peptidase 4 [EC:3.4.14.5]	g2476	0.188	2.62E-04	1.36E-03
type II pantothenate kinase [EC:2.7.1.33]	g786	0.222	2.74E-04	1.34E-03
putative endopeptidase [EC:3.4.24.-]	g6607	0.206	2.87E-04	1.32E-03
phosphotransferase system, enzyme I, PtsI [EC:2.7.3.9]	g943	0.173	2.89E-04	1.26E-03
DNA polymerase III subunit epsilon [EC:2.7.7.7]	g4232	0.195	3.10E-04	1.28E-03
7-cyano-7-deazaguanine reductase [EC:1.7.1.13]	g1148	0.162	7.13E-04	2.82E-03
chorismate mutase [EC:5.4.99.5]	g4136	0.157	7.17E-04	2.71E-03
lysozyme	g2842	0.229	7.51E-04	2.71E-03
D-alanyl-D-alanine dipeptidase [EC:3.4.13.22];D-alanyl-D-alanine dipeptidase [EC:3.4.13.-]	g2444	0.184	9.03E-04	3.12E-03
peptidyl-dipeptidase Dcp [EC:3.4.15.5]	g5103	0.168	9.07E-04	3.01E-03
phosphate butyryltransferase [EC:2.3.1.19]	g1485	0.151	9.97E-04	3.18E-03
mannose-1-phosphate guanylyltransferase [EC:2.7.7.22]	g6780	0.136	1.63E-03	5.01E-03
pyruvate carboxylase subunit B [EC:6.4.1.1]	g1706	0.129	1.76E-03	5.22E-03
Lrp/AsnC family transcriptional regulator, regulator for asnA, asnC and gidA	g4669	0.171	2.45E-03	7.01E-03
hypothetical protein	g3162	0.17	2.49E-03	6.90E-03
curved DNA-binding protein	g5998	0.106	2.55E-03	6.84E-03
GDP-L-fucose synthase [EC:1.1.1.271]	g1238	0.094	2.69E-03	6.98E-03
2-amino-4-hydroxy-6-hydroxymethyldihydropteridine pyrophosphokinase [EC:2.7.6.3];2-amino-4-hydroxy-6-hydroxymethyldihydropteridine diphosphokinase [EC:2.7.6.3]	g6519	0.136	2.78E-03	6.99E-03
manganese transport protein	g6034	0.167	3.26E-03	7.95E-03

carbamate kinase [EC:2.7.2.2]	g2715	0.123	3.58E-03	8.49E-03
PhnP protein	g6107	0.113	3.69E-03	8.51E-03
UDP-N-acetylglucosamine acyltransferase [EC:2.3.1.129]	g1040	0.144	4.97E-03	1.10E-02
topoisomerase IV subunit B [EC:5.99.1.-]	g156	0.133	5.23E-03	1.10E-02
topoisomerase IV subunit A [EC:5.99.1.-]	g4626	0.134	5.23E-03	1.10E-02
starvation-inducible DNA-binding protein	g5676	0.132	5.31E-03	1.10E-02
arylsulfatase [EC:3.1.6.1]	g3556	0.089	5.39E-03	1.10E-02
replicative DNA helicase [EC:3.6.4.12];replicative DNA helicase [EC:3.6.1.-]	g499	0.153	5.96E-03	1.20E-02
aspartate aminotransferase [EC:2.6.1.1]	g604	0.096	6.37E-03	1.20E-02
carboxyl-terminal processing protease [EC:3.4.21.102]	g5632	0.068	8.02E-03	1.50E-02
Fe-S cluster assembly protein SufB	g5827	0.079	1.00E-02	1.90E-02
type II restriction enzyme [EC:3.1.21.4]	g6230	0.053	1.10E-02	2.00E-02
levanase [EC:3.2.1.65]	g2700	0.07	1.10E-02	1.90E-02
cell division protein FtsA	g4371	0.117	1.10E-02	2.00E-02
L-asparaginase [EC:3.5.1.1]	g2597	0.075	1.20E-02	2.00E-02
rod shape determining protein RodA	g3761	0.059	1.50E-02	2.00E-02
HlyD family secretion protein	g4321	0.09	1.50E-02	2.50E-02
monovalent cation:H+ antiporter-2, CPA2 family	g3393	0.086	1.60E-02	2.40E-02
1,4-dihydroxy-2-naphthoate octaprenyltransferase [EC:2.5.1.74];1,4-dihydroxy-2-naphthoate octaprenyltransferase [EC:2.5.1.74 2.5.1.-];1,4-dihydroxy-2-naphthoate octaprenyltransferase [EC:2.5.1.-]	g2953	0.086	1.70E-02	2.50E-02
aminoacylhistidine dipeptidase [EC:3.4.13.3];dipeptidase D [EC:3.4.13.-]	g2475	0.068	2.10E-02	2.60E-02
N-carbamoylputrescine amidase [EC:3.5.1.53]	g6715	0.114	2.20E-02	2.60E-02
GMP synthase (glutamine-hydrolysing) [EC:6.3.5.2]	g4870	0.11	2.40E-02	3.10E-02
ATP-dependent DNA helicase RecQ [EC:3.6.1.-];ATP-dependent DNA helicase RecQ [EC:3.6.4.12]	g3699	0.06	2.80E-02	3.20E-02
2',3'-cyclic-nucleotide 2'-phosphodiesterase [EC:3.1.4.16]	g3247	0.082	2.80E-02	3.50E-02
phosphate:Na+ symporter;phosphate:Na+ symporter, PNaS family	g6033	0.083	3.90E-02	4.00E-02

Supplementary Table 5: Mean relative abundances of bacterial operational taxonomic units among groups (Figure 3).

	Controls (n=29)		T2DM_NP (n=21)		T2DM_P (n=28)		*P*-value Controls (n=29) vs T2DM_P (n=28)		*P*-value T2DM_NP (n=21) vs T2DM_P (n=28)	
	Mean	SD	Mean	SD	Mean	SD	T.text	Adjusted GLM[a]	T.text	Adjusted GLM[a]
Speices										
Prevotella copri	0.0619	0.061	0.1008	0.1510	0.2071	0.1925	< 0.001 *FDR*	< 0.001	0.037 *FDR*	0.043
Blautia wexlerae	0.0024	0.0029	0.0011	0.0007	0.0011	0.0006	0.022	0.012	-	-
Faecalibacterium prausnitzii	0.0772	0.0813	0.0757	0.0432	0.0504	0.0273	0.028	0.008	0.044	0.007
Coprococcus eutactus	0.0045	0.0057	0.0026	0.0023	0.0053	0.0060	0.036	0.021	-	0.022
Flavonifractor plautii	0.0019	0.0028	0.0008	0.0005	0.0012	0.0007	0.039	0.036	-	0.017
Genus										
Prevotella	0.0711	0.0636	0.1076	0.1589	0.2174	0.1955	< 0.001 *FDR*	< 0.001	0.032 *FDR*	0.043
Blautia	0.0024	0.0029	0.0011	0.0007	0.0011	0.0006	0.022	0.012	-	-
Faecalibacterium	0.0772	0.0813	0.0757	0.0432	0.0505	0.0273	0.028	0.008	0.044	0.007
Family										
Prevotellaceae	0.0752	0.0645	0.1129	0.1578	0.2278	0.2061	< 0.001 *FDR*	< 0.001	0.034 *FDR*	0.041
Fusobacteriaceae	0.0271	0.0540	0.0143	0.0367	0.0049	0.0064	0.036	0.033	-	-
Ruminococcaceae	0.1607	0.1090	0.1592	0.0850	0.1123	0.0645	0.046	0.037	0.043	0.023
Order										
Bacteroidales	0.2822	0.0978	0.3803	0.1600	0.4355	0.1328	< 0.001 *FDR*	< 0.001	-	-
Clostridiales	0.3522	0.1496	0.3402	0.1077	0.2657	0.1045	0.014 *FDR*	0.031	0.021	0.016
Fusobacteriales	0.0271	0.0540	0.0143	0.0367	0.0049	0.0064	0.036	0.033	-	-
Class										
Bacteroidia	0.2822	0.0978	0.4115	0.1400	0.4121	0.1546	< 0.001 *FDR*	< 0.001	-	-
Phylum										
Bacteroidetes	0.2822	0.0978	0.3803	0.16	0.4355	0.1328	< 0.001 *FDR*	< 0.001	-	-
Fusobacteria	0.0271	0.054	0.0143	0.0367	0.0049	0.0064	0.036	0.033	-	-

Mean relative abundances are shown as percentage values.
Adjusted GLM[a]: significance adjusted for age, sex, and acarbose use, tested using GLM.
FDR < 0.05.
Abbreviations: T2DM_P, patients with type 2 diabetes mellitus and periodontitis; T2DM_NP, patients with type 2 diabetes mellitus alone (no periodontitis); SD, standard deviation; GLM, generalized linear model.

Supplementary Table 6. Forward selection analyses of variables associated with microbiota (Figure 4A).

Variables	AIC	*F*	*P*
Number of teeth	-99.19	3.49	3E-3
CPI	-98.00	2.28	0.02
Acarbose	-97.32	1.60	0.09
Age	-97.04	1.32	0.19
Sex	-96.75	1.03	0.40
GI symptoms	-96.63	0.91	0.50
Statins	-96.62	0.90	0.51
Metformin	-96.31	0.60	0.84
Pioglitazone	-96.17	0.46	0.97

Abbreviations: AIC, Akaike information criterion; CPI, Community Periodontal Index.

Supplementary Table 7. Details on the clinical correlates of gut microbiota at genus level (Figure 4B).

Spearman's *P*	HbAlc	FBG	TG	TCHO	HDL-c	LDL-c	VLDL-c	PGE_2	LXA_4	LTB_4	TNFα	IL-6	IFN-γ	IL-17	IL-22	BALP	OC
Age	0.640	0.835	0.694	0.442	0.158	0.695	0.219	0.918	0.822	0.286	0.565	0.630	0.850	0.274	0.336	0.756	0.943
Sex	0.663	0.448	0.366	0.004	0.002	0.476	0.806	0.085	0.282	0.524	0.535	0.573	0.344	0.608	0.802	0.022	0.072
Hypertension	0.002	0.142	0.874	0.544	0.034	0.403	0.792	0.235	0.753	0.889	0.758	0.747	0.515	0.889	0.770	0.684	0.585
Duration of diabetes	0.000	0.000	0.001	0.388	0.121	0.413	0.044	0.053	0.313	0.724	0.012	0.878	0.595	0.016	0.005	0.029	0.048
CPI	0.000	0.000	0.048	0.558	0.333	0.983	0.552	0.000	0.836	0.678	0.010	0.029	0.009	0.047	0.016	0.001	0.008
Number of teeth	0.000	0.000	0.024	0.888	0.038	0.796	0.109	0.003	0.873	0.976	0.128	0.073	0.409	0.303	0.015	0.028	0.085
Dry mouth	0.599	0.718	0.610	0.902	0.140	0.510	0.917	0.030	0.601	0.155	0.297	0.047	0.110	0.018	0.309	0.677	0.436
GI symptoms	0.005	0.021	0.007	0.664	0.438	0.442	0.165	0.673	0.725	0.883	0.089	0.153	0.464	0.797	0.239	0.263	0.188

Supplementary Table 8. Details on the clinical correlates of gut microbiota at genus level (Figure 4B).

Spearman's *r*	HbAlc	FBG	TG	TCHO	HDL-c	LDL-c	VLDL-c	PGE_2	LXA_4	LTB_4	TNFα	IL-6	IFN-γ	IL-17	IL-22	BALP	OC
Age	-0.054	0.024	-0.045	0.088	0.162	-0.045	-0.141	0.012	-0.026	0.122	0.066	-0.055	0.022	0.126	-0.110	-0.036	-0.008
Sex	0.050	-0.087	0.104	-0.319	-0.339	-0.082	0.028	0.196	-0.123	0.073	-0.071	0.065	0.109	0.059	0.029	-0.260	-0.205
Hypertension	0.345	0.168	0.018	-0.070	-0.241	0.096	0.030	0.136	-0.036	-0.016	0.035	0.037	0.075	-0.016	0.034	0.047	0.063
Duration of diabetes	0.698	0.719	0.373	0.099	-0.177	0.094	0.229	0.220	-0.116	-0.041	0.283	0.018	0.061	0.271	0.318	0.247	0.224
CPI	0.592	0.401	0.225	0.067	-0.111	-0.002	0.068	0.409	0.024	0.048	0.288	0.248	0.292	0.226	0.273	0.368	0.300
Number of teeth	-0.556	-0.466	-0.255	-0.016	0.235	0.030	-0.183	-0.332	-0.018	0.004	-0.174	-0.204	-0.095	-0.118	-0.274	-0.249	-0.197
Dry mouth	-0.060	-0.042	0.059	0.014	-0.169	0.076	0.012	-0.246	-0.060	-0.163	-0.120	-0.226	-0.182	-0.268	0.117	-0.048	-0.089
GI symptoms	0.313	0.262	0.301	0.050	-0.089	0.088	0.159	0.048	-0.041	-0.017	0.194	-0.164	0.084	-0.030	0.135	0.128	0.151

Supplementary Table 9. Details on the relationships of altered gut microbiota and peripheral risk markers in elderly individuals (Figure 5G).

Spearman's r	HbAlc	FBG	TG	TCHO	HDL-c	LDL-c	VLDL-c	PGE_2	LXA_4	LTB_4	TNFα	IL-6	IFN-γ	IL-17	IL-22	BALP	OC
Blautia	-0.156	-0.255	-0.137	-0.073	0.133	-0.094	-0.067	-0.270	0.083	0.025	-0.148	-0.038	-0.043	-0.076	-0.207	-0.040	-0.027
Cloacibacillus	-0.140	-0.072	0.029	0.138	-0.035	0.129	0.177	-0.142	0.167	-0.136	0.295	-0.247	0.054	-0.125	0.239	-0.041	-0.028
Eisenbergiella	-0.168	-0.077	-0.158	-0.114	0.263	-0.269	-0.127	-0.296	-0.124	-0.057	0.187	-0.257	0.033	0.088	-0.077	-0.009	-0.038
Pseudobutyrivibrio	0.125	-0.044	-0.181	-0.003	0.001	-0.014	-0.063	-0.049	-0.014	-0.101	0.053	0.020	0.009	-0.063	-0.041	0.085	0.055
Coprobacillus	0.035	-0.098	-0.035	-0.226	-0.125	-0.221	-0.122	0.043	-0.131	-0.014	0.201	-0.086	0.054	0.128	-0.032	0.005	-0.032
Faecalibacterium	-0.031	-0.109	-0.097	-0.013	0.052	-0.077	0.063	-0.320	0.004	-0.106	-0.036	-0.044	-0.275	0.048	-0.205	-0.204	-0.197
Anaerobiospirillum	-0.166	-0.132	0.031	-0.108	-0.090	-0.070	0.076	-0.163	0.065	-0.131	-0.085	-0.297	-0.109	-0.141	0.148	0.106	0.136
Christensenella	-0.240	-0.228	-0.113	0.119	0.211	-0.066	0.018	-0.193	0.086	0.129	0.162	-0.057	-0.023	0.032	0.067	0.145	0.165
Acholeplasma	-0.039	0.071	-0.004	-0.129	-0.073	-0.030	-0.027	-0.129	0.199	-0.154	0.081	-0.205	0.238	-0.166	-0.015	-0.107	-0.079
Eubacterium	-0.080	-0.115	-0.159	-0.117	0.066	-0.173	-0.047	-0.147	0.133	-0.099	0.193	-0.137	-0.150	-0.030	0.067	-0.188	-0.207
Flavonifractor	-0.037	-0.161	-0.126	0.098	0.255	-0.059	-0.084	-0.034	-0.136	-0.039	0.173	-0.055	0.042	0.069	0.075	0.101	0.080
Odoribacter	-0.209	-0.155	-0.188	-0.021	-0.096	0.017	0.097	-0.183	0.130	-0.019	0.105	-0.116	-0.031	0.101	0.147	-0.189	-0.132
Oxalobacter	-0.273	-0.333	-0.361	-0.121	0.251	-0.108	-0.151	0.057	-0.059	-0.023	-0.098	-0.112	-0.089	-0.105	-0.073	0.045	0.074
Clostridium_XlVa	-0.182	-0.164	0.003	0.301	0.167	0.213	0.111	-0.167	0.014	0.116	0.007	-0.229	0.125	0.056	0.030	-0.083	-0.054
Anaerofilum	-0.045	-0.158	-0.252	-0.008	-0.015	0.030	-0.083	-0.193	0.037	0.037	-0.170	0.139	-0.046	0.200	-0.146	-0.176	-0.146
Fusobacterium	-0.186	-0.215	0.109	0.226	0.205	0.057	0.112	0.067	-0.011	0.083	0.091	-0.175	-0.021	-0.042	0.063	-0.024	-0.012
Collinsella	0.187	0.102	0.268	-0.103	-0.082	-0.118	0.030	-0.129	-0.076	-0.069	0.267	-0.126	-0.038	0.092	0.288	-0.121	-0.105
Parabacteroides	0.062	0.011	0.116	-0.058	0.081	-0.141	-0.004	0.026	-0.184	-0.038	0.243	-0.142	0.083	0.085	0.119	-0.099	-0.081
Alistipes	-0.070	-0.002	-0.161	0.045	0.154	-0.051	0.003	-0.208	0.065	-0.146	0.178	-0.194	0.092	0.079	0.037	-0.009	0.002
Haemophilus	0.245	0.119	0.050	0.021	-0.122	0.080	0.090	-0.039	0.010	-0.054	-0.104	0.008	0.126	0.133	0.097	0.063	0.058
Gemmiger	0.045	0.075	-0.085	-0.016	0.036	-0.023	0.018	-0.188	0.031	-0.112	-0.128	-0.020	0.025	0.075	-0.015	-0.012	-0.022
Paraprevotella	-0.035	-0.004	0.102	0.200	0.041	0.124	0.217	-0.007	0.073	-0.078	0.058	-0.319	0.143	-0.092	0.159	-0.045	-0.063
Holdemanella	0.196	0.083	-0.006	-0.174	-0.240	-0.013	-0.034	-0.039	-0.014	-0.086	0.033	0.060	0.008	0.139	0.128	-0.226	-0.201
Prevotella	0.205	0.086	0.036	0.136	-0.110	0.225	0.139	0.112	0.200	-0.035	-0.156	0.112	-0.024	-0.059	0.200	-0.022	-0.004
Aggregatibacter	0.268	0.179	0.078	0.145	-0.029	0.106	0.125	0.240	-0.126	-0.034	-0.140	0.234	-0.050	-0.023	-0.004	0.038	0.038
Sutterella	0.231	0.336	0.119	0.153	-0.134	0.222	0.034	0.094	0.086	-0.182	0.138	-0.046	0.181	0.076	0.234	0.095	0.103
Unassigned	0.094	0.226	0.036	0.020	0.092	-0.170	0.056	-0.077	0.074	0.075	0.277	0.016	0.024	0.201	0.227	0.001	-0.084
Dialister	0.199	0.326	0.096	-0.026	-0.072	-0.148	0.066	0.079	0.172	-0.068	0.248	-0.003	0.024	0.075	0.119	0.210	0.173
Howardella	0.114	0.027	-0.035	-0.107	-0.152	0.003	0.030	0.129	0.177	-0.098	0.051	0.157	-0.168	-0.114	0.151	-0.032	-0.015
Streptococcus	0.176	0.038	-0.089	-0.015	-0.044	0.018	0.016	0.137	-0.008	0.093	-0.136	0.136	0.167	0.093	0.039	0.116	0.081
Alloprevotella	0.140	0.060	-0.096	-0.201	-0.098	-0.106	-0.155	0.119	0.040	0.003	0.056	0.035	0.032	0.027	0.094	0.171	0.144
Bacteroides	-0.034	0.037	0.068	-0.004	0.087	-0.158	0.026	-0.074	-0.056	0.080	0.133	-0.176	0.183	0.114	0.025	0.108	0.061
Veillonella	0.275	0.193	-0.025	0.071	-0.064	0.059	0.049	0.115	-0.055	-0.053	-0.154	0.203	0.212	0.251	-0.008	0.125	0.073
Dorea	0.150	-0.005	0.054	0.142	-0.019	0.148	0.116	0.055	0.063	-0.072	-0.018	0.082	-0.225	-0.020	0.069	0.027	0.058

Supplementary Table 10. Details on the relationships of altered gut microbiota and peripheral risk markers in elderly individuals (Figure 5G).

Spearman's *P*	HbAlc	FBG	TG	TCHO	HDL-c	LDL-c	VLDL-c	PGE_2	LXA_4	LTB_4	TNFα	IL-6	IFN-γ	IL-17	IL-22	BALP	OC
Blautia	0.173	0.024	0.232	0.527	0.244	0.413	0.559	0.017	0.467	0.829	0.197	0.739	0.707	0.510	0.068	0.726	0.811
Cloacibacillus	0.221	0.528	0.804	0.227	0.764	0.261	0.120	0.214	0.144	0.236	0.009	0.029	0.637	0.275	0.035	0.725	0.805
Eisenbergiella	0.143	0.502	0.168	0.320	0.020	0.017	0.270	0.008	0.279	0.622	0.100	0.023	0.774	0.445	0.500	0.937	0.743
Pseudobutyrivibrio	0.276	0.705	0.113	0.979	0.994	0.901	0.581	0.668	0.900	0.379	0.643	0.863	0.938	0.586	0.720	0.462	0.630
Coprobacillus	0.759	0.393	0.762	0.047	0.276	0.052	0.287	0.708	0.254	0.901	0.078	0.452	0.638	0.266	0.782	0.964	0.780
Faecalibacterium	0.787	0.341	0.398	0.911	0.651	0.505	0.585	0.004	0.972	0.354	0.756	0.703	0.015	0.674	0.071	0.073	0.084
Anaerobiospirillum	0.147	0.248	0.785	0.346	0.431	0.541	0.508	0.154	0.572	0.254	0.457	0.008	0.344	0.217	0.197	0.357	0.237
Christensenella	0.035	0.045	0.326	0.298	0.064	0.567	0.874	0.091	0.455	0.261	0.155	0.619	0.844	0.783	0.560	0.205	0.148
Acholeplasma	0.736	0.539	0.974	0.260	0.528	0.796	0.811	0.259	0.081	0.179	0.482	0.072	0.036	0.146	0.894	0.351	0.493
Eubacterium	0.486	0.315	0.165	0.309	0.566	0.130	0.680	0.199	0.244	0.390	0.090	0.231	0.189	0.793	0.557	0.100	0.069
Flavonifractor	0.746	0.160	0.272	0.394	0.025	0.611	0.465	0.771	0.236	0.734	0.130	0.632	0.717	0.550	0.515	0.380	0.485
Odoribacter	0.067	0.174	0.099	0.852	0.402	0.880	0.400	0.108	0.257	0.871	0.360	0.313	0.789	0.378	0.200	0.097	0.250
Oxalobacter	0.016	0.003	0.001	0.292	0.027	0.348	0.188	0.618	0.605	0.843	0.393	0.329	0.438	0.360	0.527	0.697	0.518
Clostridium_XlVa	0.111	0.150	0.979	0.007	0.144	0.061	0.332	0.145	0.906	0.311	0.949	0.044	0.275	0.626	0.792	0.470	0.640
Anaerofilum	0.695	0.168	0.026	0.944	0.897	0.794	0.468	0.090	0.748	0.750	0.136	0.224	0.692	0.079	0.203	0.122	0.202
Fusobacterium	0.103	0.059	0.341	0.047	0.072	0.620	0.329	0.559	0.924	0.471	0.430	0.126	0.857	0.718	0.582	0.836	0.917
Collinsella	0.102	0.372	0.018	0.368	0.473	0.303	0.792	0.259	0.511	0.550	0.018	0.271	0.744	0.423	0.011	0.291	0.360
Parabacteroides	0.591	0.923	0.312	0.614	0.480	0.219	0.974	0.819	0.108	0.741	0.032	0.214	0.471	0.460	0.299	0.388	0.479
Alistipes	0.540	0.987	0.159	0.696	0.177	0.655	0.976	0.068	0.573	0.201	0.118	0.089	0.424	0.491	0.749	0.936	0.985
Haemophilus	0.030	0.299	0.667	0.854	0.289	0.486	0.434	0.736	0.928	0.636	0.365	0.948	0.272	0.246	0.399	0.583	0.612
Gemmiger	0.697	0.512	0.458	0.892	0.753	0.845	0.873	0.100	0.789	0.329	0.263	0.862	0.828	0.516	0.893	0.919	0.846
Paraprevotella	0.758	0.976	0.373	0.079	0.722	0.279	0.057	0.952	0.523	0.498	0.615	0.004	0.213	0.423	0.165	0.696	0.584
Holdemanella	0.085	0.468	0.960	0.128	0.034	0.913	0.769	0.735	0.906	0.456	0.772	0.602	0.946	0.226	0.263	0.046	0.077
Prevotella	0.071	0.452	0.756	0.235	0.338	0.048	0.224	0.328	0.079	0.760	0.173	0.329	0.836	0.610	0.080	0.845	0.975
Aggregatibacter	0.018	0.116	0.500	0.206	0.800	0.355	0.275	0.035	0.272	0.766	0.222	0.040	0.663	0.840	0.969	0.741	0.740
Sutterella	0.042	0.003	0.300	0.180	0.241	0.051	0.765	0.412	0.456	0.111	0.227	0.688	0.113	0.506	0.039	0.409	0.369
Unassigned	0.413	0.047	0.752	0.862	0.423	0.137	0.628	0.503	0.518	0.515	0.014	0.886	0.838	0.077	0.046	0.991	0.467
Dialister	0.080	0.004	0.403	0.824	0.531	0.197	0.567	0.493	0.133	0.556	0.029	0.977	0.836	0.512	0.301	0.065	0.130
Howardella	0.319	0.812	0.761	0.351	0.184	0.978	0.797	0.261	0.120	0.391	0.657	0.171	0.140	0.322	0.188	0.780	0.893
Streptococcus	0.124	0.743	0.439	0.900	0.701	0.875	0.888	0.231	0.947	0.420	0.234	0.237	0.145	0.417	0.732	0.313	0.482
Alloprevotella	0.222	0.601	0.403	0.078	0.394	0.355	0.175	0.299	0.726	0.977	0.629	0.761	0.782	0.813	0.415	0.134	0.207
Bacteroides	0.769	0.748	0.553	0.973	0.451	0.166	0.820	0.517	0.627	0.485	0.247	0.122	0.110	0.322	0.828	0.347	0.596
Veillonella	0.015	0.090	0.831	0.537	0.577	0.607	0.669	0.317	0.632	0.645	0.179	0.075	0.062	0.027	0.944	0.274	0.524
Dorea	0.189	0.967	0.641	0.216	0.868	0.196	0.311	0.632	0.582	0.533	0.878	0.473	0.047	0.863	0.550	0.817	0.614

Supplementary Table 11. Diabetes bowel symptom questionnaire.

**Part A**	
**DO YOU HAVE:**	**HOW SERIOUS (1-5 points):**
1. Abdominal pain	________
2. Pain better with bowel action	________
3. Pain after meals	________
4. Pain before meals	________
5. Pain better with milk	________
6. Hard stools	________
7. Loose stools	________
8. Faecal urgency	________
9. Straining	________
10. Incomplete evacuation	________
11. Flatus incontinence	________
12. Amount of Abdominal pain	________
13. Pain woke from sleep	________
**DO YOU HAVE:**	**HOW OFTEN (0-6 points):**
14. More than 3 bowel actions per day	________
15. Less than 3 bowel actions per week	________
16. Manual assistance with defecation	________
17. Anal blockage	________
18. Amount of faecal incontinence	________
19. Manual assistance with defecation	________
20. Amount of faecal incontinence	________
21. Change in bowel habit	________
22. Abdominal or bowel disease	________
23. GI surgery	________
**Part B**	
**DO YOU HAVE:**	**HOW SERIOUS (1-5 points):**
24. Acid regurgitation	________
25. Dysphagia	________
26. Early satiety	________
27. Vomiting	________
28. Persistence of food in stomach	________
29. Abdominal bloating	________
30. Abdominal distension	________
31. Loss of appetite	________
32. Nausea	________
33. Retching	________
34. Heartburn	________
